# The risks and rewards of covariate adjustment in randomized trials: an assessment of 12 outcomes from 8 studies

**DOI:** 10.1186/1745-6215-15-139

**Published:** 2014-04-23

**Authors:** Brennan C Kahan, Vipul Jairath, Caroline J Doré, Tim P Morris

**Affiliations:** 1 Pragmatic Clinical Trials Unit, Queen Mary University of London, London E1 2AB, UK; 2 Nuffield Department of Medicine, University of Oxford, Oxford OX3 9DU, UK; 3 Comprehensive Clinical Trials Unit, University College London, London WC1E 6BT, UK; 4 MRC Clinical Trials Unit, University College London, London WC2B 6NH, UK

**Keywords:** Adjusted analysis, clinical trial, covariate adjustment, power, randomized controlled trial, regression

## Abstract

**Background:**

Adjustment for prognostic covariates can lead to increased power in the analysis of randomized trials. However, adjusted analyses are not often performed in practice.

**Methods:**

We used simulation to examine the impact of covariate adjustment on 12 outcomes from 8 studies across a range of therapeutic areas. We assessed (1) how large an increase in power can be expected in practice; and (2) the impact of adjustment for covariates that are not prognostic.

**Results:**

Adjustment for known prognostic covariates led to large increases in power for most outcomes. When power was set to 80% based on an unadjusted analysis, covariate adjustment led to a median increase in power to 92.6% across the 12 outcomes (range 80.6 to 99.4%). Power was increased to over 85% for 8 of 12 outcomes, and to over 95% for 5 of 12 outcomes. Conversely, the largest decrease in power from adjustment for covariates that were not prognostic was from 80% to 78.5%.

**Conclusions:**

Adjustment for known prognostic covariates can lead to substantial increases in power, and should be routinely incorporated into the analysis of randomized trials. The potential benefits of adjusting for a small number of possibly prognostic covariates in trials with moderate or large sample sizes far outweigh the risks of doing so, and so should also be considered.

## Background

Adjustment for baseline covariates in the analysis of randomized controlled trials (RCTs) can lead to a substantial increase in power when the covariates are highly prognostic [1-10]. Hernandez *et al.* found that increases in power of over 20% are possible in certain circumstances [3], and this has been demonstrated in simulation studies based on real datasets [1,2,6] and confirmed through reanalysis of an RCT [8]. Other benefits of adjustment include protection against chance imbalances in important baseline covariates [7], and maintaining correct type I error rates when the covariates have been used in the randomization process [11-14].

Despite these benefits, unadjusted analyses dominate in practice; reviews have found that between 24 and 34% of trials use covariate adjustment for their main analysis [15-19]. It is unclear why so few RCTs perform adjusted analyses. Researchers may be unsure or sceptical as to how much of an increase in power is likely to occur in practice. Few articles have examined this issue using real data, and most have been limited to datasets in traumatic brain injury [2,5,8] or Alzheimer’s disease [20]. Further research to assess the potential increase in power through adjustment for known prognostic factors, and the decrease in power through adjustment for nonprognostic factors, would allow researchers to make more informed decisions as to whether covariate adjustment is likely to be worthwhile in their own trial.

In this paper we give an overview of the benefits and risks of covariate adjustment in RCTs, using examples from a previously published trial. We then examine 12 outcomes from 8 different studies in a variety of disease areas to determine (1) the likely increase in power through adjustment for known prognostic covariates; and (2) the likely decrease in power through inadvertently adjusting for nonprognostic covariates.

## Methods

### Benefits of covariate adjustment

We describe some of the benefits of covariate adjustment next, and illustrate these concepts using data from the Second Multi-centre Intra-pleural Sepsis Trial (MIST2) [21]. MIST2 was a four-arm trial that compared tissue plasminogen activator (tPA), DNase and tPA + DNase against placebo in patients with a pleural effusion. We focus here on the treatment comparison between the tPA + DNase and placebo groups, for simplicity. The primary outcome measure was the change from baseline to day 7 in the size of the patient’s pleural effusion (a continuous outcome). A major secondary outcome measure was the need for surgery at 90 days (a binary outcome). Patients were randomized using minimization [22-25]; stratification factors were: the size of the pleural effusion at baseline; whether the infection was hospital-acquired, and the presence of purulent pleural fluid.

#### Increase in power (continuous outcomes)

One of the main advantages of covariate adjustment is that it can lead to increased power. For continuous outcomes, this occurs because the covariates help to explain some of the variation in outcomes between patients, leading to smaller standard errors (SEs) for the treatment effect. The amount by which the SE is reduced depends on the correlation between the covariates and the outcome; the higher the correlation, the larger the increase in power [4].

This is particularly relevant for continuous outcomes that are also measured at baseline (for example, a pain score might be measured at baseline and again at 6 months). These baseline measurements are generally highly correlated with outcome, and so adjustment can lead to substantial gains in power [7,26]. This is true regardless of whether the outcome measure is defined as the measurement at 6 months, or as a change from baseline to 6 months; as long as the analysis adjusts for the baseline measurement, both analyses provide identical treatment effect estimates and SEs.

In the MIST2 trial, the correlation between the size of the patient’s pleural effusion at baseline and day 7 was 0.44. Accounting for baseline effusion size in the analysis resulted in a large reduction in the SE of the treatment effect (unadjusted SE of 4.3 vs adjusted SE of 2.8; a 35% reduction), leading to a substantial increase in power.

#### Increase in power (binary and time-to-event outcomes)

Adjusting for important prognostic covariates in the analysis of a binary or time-to-event outcome when estimating an odds ratio or hazard ratio will also lead to an increase in power. However, unlike continuous outcomes, where adjusting for important covariates leads to a reduction in the SE, adjusting for important covariates with a binary or time-to-event outcome will generally lead to larger SEs; however, this increase in the SE is offset by an increase in the estimated treatment effect; that is, estimated odds or hazard ratios will be further from 1 (where an odds or hazard ratio of 1 indicates no treatment effect), assuming that there is a true treatment effect. Therefore, adjustment for covariates with a binary or time-to-event outcome will generally lead to a loss in precision (wider confidence intervals) but increased power [9,10].

This difference occurs because the methods are estimating different treatment effects. Adjusted analyses lead to subject-specific (or conditional) estimates, which compare an ‘intervention’ patient with a ‘control’ patient with the same covariates. Unadjusted analyses lead to marginal (or population-averaged) estimates, which compare an ‘intervention’ patient with a ‘control’ patient who has been randomly selected from the trial, regardless of their covariate values. For continuous outcomes, subject-specific and marginal analyses have the same expected treatment effect, but this is not generally the case for binary and time-to-event outcomes [9].

In the MIST2 trial, adjustment for the size of the pleural effusion at baseline in the analysis of surgery at 90 days led to increases in both the size of the treatment effect and its SE: an unadjusted log(odds ratio) of -1.14 (SE, 0.84) vs an adjusted value of -1.46 (SE, 0.87). However, because the increase in the treatment effect through adjustment was much higher than the increase in the SE, this led to a substantial increase in the *Z* statistic (unadjusted -1.36 vs adjusted -1.67; a 23% increase), leading to increased power.

#### Protection against chance imbalance in important baseline covariates

Randomization ensures that, on average, both known and unknown covariates are well balanced between treatment groups [23]. However, randomization does not guarantee balance; in any individual trial, there may be large imbalances in important prognostic covariates between treatment groups merely by chance. Any such imbalance can give an unfair advantage to one treatment group over another if not accounted for in the analysis. Therefore, prespecifying that important baseline covariates are included in the analysis will help to ensure that any chance imbalances between treatment groups in these covariates will not affect treatment effect estimates [27].

In the MIST2 trial, there was an imbalance between treatment arms in the size of the pleural effusion at baseline (mean placebo 39 (standard deviation (SD), 22) vs mean tPA + DNase 47 (SD 24)). Because the size of the effusion at baseline was highly correlated with the size at day 7 (0.44), this imbalance gave an unfair advantage to the tPA + DNase group. The unadjusted treatment effect was -12.3, but was reduced to -7.6 after adjustment (a 38% reduction).

#### Appropriate confidence intervals and P values after stratified randomization

Many trials use stratified randomization to balance key prognostic covariates between treatment arms. A recent review found 63% of trials used at least one stratification factor; however, only 26% of these appropriately accounted for these variables in their primary analysis [12]. If the stratification factors are associated with outcome, then stratified randomization has the effect of forcing the outcomes between treatment groups to be more similar than they otherwise would be. This leads to correlation between the treatment groups, which violates the standard statistical assumption of independence. If this correlation is ignored (by not adjusting for the stratification factors in the analysis) then the SE for treatment effect will be biased upwards, leading to confidence intervals that are too wide, *P* values that are too large, incorrect type I error rates and a reduction in power. Conversely, accounting for the stratification factors in the analysis leads to correct SEs and no loss in power [11-14,28]. Therefore, it is essential that stratification factors be accounted for in the trial analysis.

The MIST2 trial used three stratification factors in the randomization process. Previous research has shown that not accounting for these stratification factors in the analysis led to SEs that were biased upwards by 14 to 15%, which in turn led to type I error rates of around 2.6% (rather than the nominal 5%). This resulted in major reductions in power (adjusted 80% vs unadjusted 59%) [11].

### Risks of covariate adjustment

#### Loss in power due to adjustment for nonprognostic covariates

Although adjustment for prognostic covariates can lead to increased power, adjustment for nonprognostic covariates can lead to increased SEs, and thus a decrease in power. This occurs because each continuous or binary baseline covariate uses a ‘degree of freedom’, which effectively reduces the sample size, meaning that there is less information with which to estimate the treatment effect (in cases where the covariate actually is prognostic, the benefits of the prognostic ability outweigh any loss of information, and power will be increased despite the loss of a degree of freedom). This is particularly an issue with small sample sizes (as reducing the effective sample size from 50 to 40 patients through adjustment for 10 nonprognostic covariates will have a much larger impact than reducing it from 500 to 490 patients). Therefore, caution is required in the number of covariates that are included in the analysis if the sample size is small.

#### Inflation of the type I error rate due to overstratification

Covariate adjustment can lead to inflated type I error rates (that is, increased probability of a false positive) when there is a small sample size and a binary or time-to-event outcome [11,29]. This is because covariate adjustment can lead to overstratification in these situations, meaning that there are too many covariates in relation to the number of observed events. It is therefore important to keep the overall sample size and expected event rate in mind when deciding how many covariates to include in the analysis.

#### Missing data on covariates

If some patients are missing data on certain covariates that were to be included in the analysis, it may be unclear how to proceed. Two unsatisfactory options are to perform a complete case analysis (where patients with missing values for the covariates are excluded from the analysis) and to exclude covariates with missing data from the analysis. A complete case analysis is unsatisfactory as it will reduce the sample size, and therefore reduce power (the opposite of our intention). Excluding covariates with missing data from the analysis is similarly unsatisfactory, as it deviates from the prespecified analysis plan, and might result in key prognostic covariates being excluded, negating some of the benefits of adjustment.

A preferable and simple alternative is to use mean imputation [30], where the missing values are replaced with the mean of the observed data. This has been shown to give unbiased estimates of treatment effect and preserve the type I error rate in RCTs (unlike in observational studies, where it can lead to bias). This allows all patients to be included in the analysis, and should therefore increase power compared with a complete case analysis, or one that excludes the covariate. Other simple and appropriate methods of dealing with missing baseline data are also available [30].

#### Bias due to data-driven methods of choosing covariates

Methods are available to identify which covariates to include in the analysis, such as stepwise selection, where variables with large *P* values are removed, or by adjusting for covariates with a large observed difference between treatment arms at baseline. Reviews have found that between 16% and 31% of trials use these methods [15,17,18]. However, these methods use the trial data to decide which covariates to include, and have been shown to lead to incorrect type I error rates in many situations [31]. Prespecifying which variables will be included in the analysis in the protocol or analysis plan will avoid bias, and give more credibility to the trial results.

### Simulation study

We performed a simulation study to assess the increase or decrease in power from covariate adjustment across a number of outcomes and studies in a variety of different disease areas.

We performed simulations for 12 different outcomes (four continuous, six binary, two time-to-event) based on 8 different studies. The studies were the AUGIB study [32-35], the Function After Spinal Treatment, Exercise, and Rehabilitation (FASTER) trial [36], the MIST2 trial [21], the MOSAIC trial [37], the primary biliary cirrhosis (PBC) trial [38], the PROGRAMS trial [39], the RE01 trial [40] and the TIME2 [41] trial. Further information on each study is available in Table 1 and in Additional file 1.

**Table 1:** Description of studies

**Study**	**Disease area**	**Study type**	**Sample size**	**Outcome measure**	**Outcome type**	**Prognostic covariates**
AUGIB	Acute upper gastrointestinal bleeding	Observational study	600^a^	Mortality in hospital	Binary	(1) clinical Rockall score
				Further bleeding in hospital	Binary	(1) clinical Rockall score
				RBC transfusion in hospital	Binary	(1) presence of shock; (2) haemoglobin concentration at baseline
FASTER	Postoperative rehabilitation	RCT	316	Oswestry disability index	Continuous	(1) Oswestry disability index at baseline; (2) type of surgery
MIST2	Malignant pleural effusion	RCT	210	Size of the patient’s pleural effusion at 7 days	Continuous	(1) size of the pleural effusion at baseline; (2) hospital-acquired infection; (3) large tube size; (4) drain present
				Need for surgery at 90 days	Binary	(1) size of the pleural effusion at baseline; (2) large tube size
MOSAIC	Sleep apnoea	RCT	391	Epworth Sleepiness Score	Continuous	(1) Epworth Sleepiness Score at baseline; (2) sex; (3) MRI received at baseline
PBC	Primary biliary cirrhosis	RCT	312	Time to death	Time-to-event	(1) age; (2) albumin concentration; (3) bilirubin concentration; (4) histological stage
PROGRAMS	Extremely preterm, small for gestational age neonates	RCT	280	Sepsis-free survival up to day 14	Binary	(1) gestational age at birth; (2) birth weight
				Mortality up to day 14	Binary	(1) gestational age at birth; (2) birth weight
RE01	Metastatic renal carcinoma	RCT	347	Time to death	Time-to-event	(1) WHO score; (2) tumour grade; (3) white cell count
TIME2	Malignant pleural effusion	RCT	106	Mean breathlessness over 42 days	Continuous	(1) breathlessness at baseline; (2) performance status; (3) mesothelioma

^a^This dataset contained 6,750 patients; however, when assessing this study, we set the sample size to 600 patients to make this more realistic to a trial setting. We chose 600 as this was the median sample size found among trials with a binary primary outcome in a recent review [12]. RCT, randomized controlled trial.

Full details of the simulation study can be found in Additional file 1. Briefly, we simulated 5,000 datasets for each outcome and the simulated data were based on parameter estimates obtained from the study datasets. We used two different treatment effects; one was calculated to give 50% power (referred to as an ‘underpowered’ trial) and the other to give 80% power (an ‘adequately powered’ trial), based on an unadjusted analysis. We used between one and four known prognostic covariates for each outcome, taken from the study datasets.

For each outcome, we compared power between four different methods of analysis: (1) unadjusted for all baseline covariates; (2) adjusted for known prognostic covariates; (3) adjusted for three ‘random-noise’ covariates (which were not related to the outcome); and (4) adjusted for both known prognostic and ‘noise’ covariates. We assessed the impact of included noise covariates to determine how much of a loss in power to expect from adjusting for covariates that were not related to outcome. All analyses were performed using a regression model (linear regression for continuous outcomes, logistic regression for binary outcomes and a Cox model for time-to-event outcomes). Adjusted analyses were performed by including the covariates in the regression model. All covariates were kept in the model, regardless of statistical significance; this was to reflect adherence to a predefined analysis plan.

## Results

**Figure 1:**
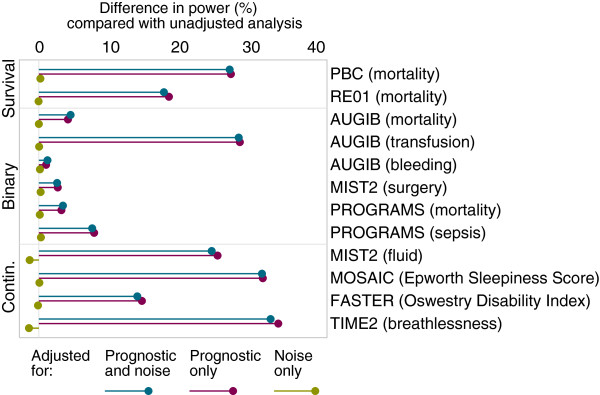
**Simulation results for ‘underpowered’ trials.** Change in power through covariate adjustment as compared with unadjusted analysis for ‘underpowered’ trials (where an unadjusted analysis gives 50% power).

**Figure 2:**
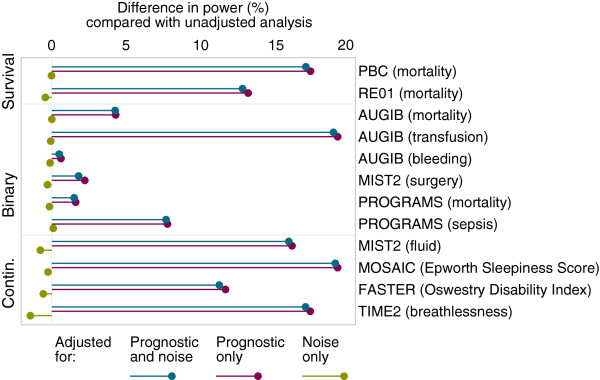
**Simulation results for ‘adequately powered’ trials.** Change in power through covariate adjustment as compared with unadjusted analysis for ‘adequately powered’ trials (where an unadjusted analysis gives 80% power).

Results are shown in Figures 1 and 2. Adjustment for prognostic covariates led to substantial increases in power for most outcomes. For ‘underpowered’ trials, covariate adjustment led to a median increase in power across the 12 outcomes from 50% to 66.7% (range 51.0 to 84.4%). Power was increased to over 55% for 8 of 12 outcomes, and to over 75% for 5 of 12 studies. The increase in power from covariate adjustment was smaller in ‘adequately’ powered trials, though still substantial. The median increase in power was from 80% to 92.6% (range 80.6 to 99.4%), and power was increased to over 85% and 95% for 8 of 12 and 5 of 12 outcomes, respectively.

Adjustment for ‘noise’ covariates had little impact on power. For ‘underpowered’ trials, the largest decrease in power was from 50% to 48.6% (range 48.6% to 50.3%), and only 2 of 12 trials had a decrease in power to less than 49%. For ‘adequately’ powered trials the largest decrease in power was from 80% to 78.5% (range 78.5% to 80.1%), and only 1 of 12 trials had a decrease in power to less than 79%.

## Discussion

Although it is well known that adjustment for prognostic covariates can lead to increased power in RCTs, there has been little research attempting to quantify how much of a gain is possible under real trial conditions, or how much of a loss in power can be expected after adjustment for nonprognostic (or ‘noise’) covariates. A better understanding of this would help researchers to select appropriate covariates to adjust for in their own trials *a priori*.

In this simulation study using real patient data from a number of differing disease and therapeutic areas, we found that adjustment for strong prognostic covariates led to substantial increases in power in the majority of scenarios we studied. We additionally found that adjustment for ‘noise’ covariates had little negative impact on power. These two findings suggest that known prognostic covariates should be included in the analysis, in order to increase power. Additionally, a small number of covariates that are suspected (but not known) to be prognostic could also be included in the analysis, since the potential gains in power if they truly are prognostic far outweigh any potential loss in power if they are not prognostic. As discussed elsewhere, the covariates to be adjusted for should be prespecified in the protocol or analysis plan prior to examining the data [42].

The one exception to these recommendations is when there is a small sample size and a binary or time-to-event outcome, as adjustment for covariates in these scenarios could potentially inflate the type I error rate [11,29]. This is unlikely to be a problem for trials with a moderate or large sample size. However, it is often difficult to define how small is too small in terms of sample size; if in doubt, methods to account for prognostic covariates with a small sample size have been proposed [29].

Our study has some limitations. First, we only assessed the impact of adjusting for three ‘noise’ covariates. We chose this number, as we felt that an analysis that adjusts for a small number of covariates is generally viewed more favourably than an analysis adjusting for a large number of covariates [43]. Further research to examine the impact of adjustment for more than three suspected prognostic covariates might be useful. Second, we have not discussed the different methods of accounting for prognostic covariates that can be used. However, these issues have been discussed elsewhere [28,29,44,45], and are beyond the scope of this study. Finally, the AUGIB study was observational, and the participants might have been more heterogeneous than in most randomized trials, which could increase the apparent effect of covariate adjustment. Therefore, results from the AUGIB study should be interpreted cautiously.

## Conclusions

Researchers should adjust for known prognostic covariates. The possible benefits of also adjusting for a small number of suspected prognostic covariates with moderate or large sample sizes far outweigh any risks, and so should also be considered.

## Abbreviations

FASTER: Function After Spinal Treatment, Exercise, and Rehabilitation; MIST2: Second Multi-centre Intra-pleural Sepsis Trial; PBC: primary biliary cirrhosis; RCT: randomized controlled trial; SD: standard deviation; SE: standard error; tPA: tissue plasminogen activator.

## Competing interests

The authors declare that they have no competing interests.

## Authors’ contributions

BCK: conception and design, statistical analysis, interpretation of results and manuscript writing. CJD and TPM: study design, interpretation of results and critical revision of the manuscript. VJ: interpretation of results, and critical revision of the manuscript. All authors read and approved the final manuscript.

## Supplementary Material

Additional file 1 **Study information, simulation details and results.** Additional information on each study used, how the simulation study was performed and simulation results. Click here for file
