# Supplementary material for: The risks and rewards of covariate adjustment in randomized trials: an assessment of 12 outcomes from 8 studies
Source: Trials. 2014 Apr 23;15:139. doi: 10.1186/1745-6215-15-139 (PMC4022337; doi:10.1186/1745-6215-15-139)
Supplement: Additional file 1 — Study information, simulation details and results. Additional information on each study used, how the simulation study was performed and simulation results. [file 1745-6215-15-139-S1.docx]

**Supplementary material**

**Study descriptions**

*MIST2 dataset*

MIST2 was a four-arm randomised trial which compared tPA, DNase, and tPA + DNase against placebo in 210 patients with a pleural effusion. We performed simulations for two outcomes; the first was change from baseline to day 7 in the size of the patient’s pleural effusion (a continuous outcome), and the second was need for surgery at 90 days (a binary outcome). For the first outcome (pleural effusion) we used four baseline prognostic factors: (1) the size of the pleural effusion at baseline; (2) whether the infection was hospital-acquired or not; (3) whether a large tube size was used; and (4) whether a drain was present. For the second outcome (surgery) we used two baseline prognostic factors: (1) the size of the pleural effusion at baseline; and (2) whether a large tube size was used.

*TIME2 dataset*

TIME2 was a randomised trial which compared an indwelling pleural catheter with a chest tube and talc slurry pleurodesis to relieve breathlessness in 106 patients with a malignant pleural effusion. We performed simulations based on the primary outcome of mean breathlessness (based on a visual analog scale) over the first 42 days after randomisation (a continuous outcome). We used three baseline prognostic factors; (1) breathlessness at baseline; (2) performance status (0-1 vs 2-3); and (3) mesothelioma vs non-mesothelioma.

*MOSAIC dataset*

MOSAIC was a randomised trial which compared continuous positive airway pressure vs standard care in 391 patients with minimally symptomatic obstructive sleep apnoea. We performed simulations based on the primary outcome, which was the Epworth Sleepiness Score (a continuous outcome). We used three baseline prognostic factors; (1) the Epworth Sleepiness Score at baseline; (2) gender; and (3) whether the patient received an MRI at baseline.

*FASTER dataset*

FASTER was a 2x2 factorial trial comparing rehabilitation vs no rehabilitation and an educational booklet vs no educational booklet in 316 patients following back surgery. We performed simulations based on the primary outcome, which was the Oswestry disability index (a continuous outcome). We used two baseline prognostic factors: (1) the Oswestry disability index at baseline; and (2) whether the surgery was discectomy for a herniated disc, or decompression for spinal stenosis.

*AUGIB dataset*

The AUGIB dataset was an observational study, which contained data gathered as part of a UK nationwide audit of patients with acute upper gastrointestinal bleeding. We performed simulations for three different outcomes (all binary); the first was in-hospital mortality, the second was further bleeding while in hospital, and the third was whether the patient received a red blood cell transfusion while in hospital. The audit collected data on 6750 patients; however we set the sample size to 600 patients for simulations (as this was the median sample size found among trials with a binary primary outcome in a recent review [[1](#_ENREF_1)]). For the outcomes of mortality and further bleeding, we used the clinical Rockall score (a commonly used risk score which is highly associated with mortality) as the only baseline prognostic factor. For the red blood cell transfusion outcome, we used two baseline prognostic factors; (1) presence of shock; and (2) haemoglobin concentration at baseline.

It should be noted that a re-analysis of the AUGIB dataset itself would not be useful in assessing the issue of covariate adjustment (as there is likely to be confounding in this observational dataset, making it impossible to determine which changes in treatment effects and standard errors were due to adjustment for confounding, and which were due to adjustment more generally). However, this issue does not occur when using the AUGIB dataset as a basis for simulation, as patients in each simulated dataset are randomised to treatment, and so there is no issue with confounding.

*PROGRAMS dataset*

PROGRAMS was a randomised trial comparing granulocyte-macrophage colony stimulating factor with standard care in 280 preterm neonates. We performed simulations for two binary outcomes; the first was sepsis-free survival up to day 14 (the primary outcome), and the second was mortality up to day 14. We used two baseline prognostic factors for each outcome; (1) the gestational age at birth; and (2) birth weight.

*PBC dataset*

PBC was a randomised trial comparing D-penicillamine vs placebo in 312 patients with primary biliary cirrhosis. We performed simulations based on the primary outcome of mortality (a time-to-event outcome). We used four baseline prognostic factors; (1) patient age; (2) albumin; (3) the log transformed value of bilirubin; and (4) the patient’s histological stage (1-4).

*RE01 dataset*

RE01 was a randomised trial which compared interferon-α vs medroxyprogesterone acetate in 347 patients with metastatic renal carcinoma. We performed simulations based on the outcome of mortality (a time-to-event outcome). We used three baseline prognostic factors: (1) a WHO score of 0/1 vs 2; (2) a tumour grade of 1–3 vs 4; and (3) a log transformation of the white cell count.

**Simulation study**

We performed a simulation study for each of the 12 outcomes to assess the increase in power through adjustment for known prognostic factors, as well as the decrease in power through adjustment for three non-prognostic factors.

We generated continuous outcomes from the following model:

$$Y_{i}=\alpha+\beta_{treat}X_{treat}+\beta_{adj}X_{adj}+\varepsilon_{i}$$

where Y_i_ is the outcome for the i^th^ patient, *β*_treat_ and *β*_adj_ are the effects of treatment and the baseline covariates respectively, X_treat_ and X_adj_ denote whether the patient received treatment or not, and the level of the covariate respectively, and ε_i_~N(0, σ^2^).

We generated binary outcomes from the following model:

$$Y_{i}^{*}=\alpha+\beta_{treat}X_{treat}+\beta_{adj}X_{adj}+\varepsilon_{i}$$

where Y_i_^*^ is a latent outcome for the *i*th patient, *β*_treat_ is the log odds ratio for treatment, *β*_adj_ is the log odds ratios of the baseline covariates, and ε_i_ is a random error term that follows a logistic distribution with mean 0 and variance π^2^/3. Binary responses were generated as Y_i_ = 1 if Y_i_^*^>0, and 0 otherwise.

We generated time to event outcomes using the method described by Bender *et al* [[2](#_ENREF_2)]:

$$Y_{i}=H_{0}^{-1}[-\ln(U)\exp(-\beta_{treat}X_{treat}-\beta_{adj}X_{adj})]$$

where *Y_i_* is the time to death, *β*_treat_ is the log hazard ratio for treatment, *β*_adj_ is the log hazard ratio of the baseline covariates, *H_0_* is the cumulative baseline hazard function, and *U*~Uniform(0, 1). This model implies proportional hazards.

We generated covariates (X_adj_) using a multivariate normal distribution. We used a covariance matrix estimated from the original dataset to preserve the correlation structure between covariates in the simulated datasets. We used specified cut-points for categorical variables to ensure the correct proportion of patients was in each category. We estimated the mean and standard deviation of continuous covariates, and the proportion of patients in each category for categorical variables, from the original study dataset.

We estimated the values of *β*_treat_, *β*_adj_, σ^2^, α, and *H_0_* for each outcome from the original study data using a linear regression model for continuous outcomes, a logistic regression model for binary outcomes, and a parametric survival model using a Weibull distribution for time-to-event outcomes. For time-to-event outcomes we estimated the amount of censoring from the study data and added this to each simulated dataset. We used two values of *β*_treat_ for each outcome; the first was selected to give 50% power under an unadjusted analysis, the second was selected to give 80% power under an unadjusted analysis. We used 5000 replications for each simulation scenario. Patients were randomised to one of the two treatment arms using simple randomisation.

For each outcome we compared four different methods of analysis in terms of power; (1) unadjusted for all baseline covariates; (2) adjusted for a set of known prognostic factors; (3) adjusted for a set baseline variables which are unrelated to outcome (noise variables); and (4) adjusted for both known prognostic factors and a noise variables. We assessed the impact of included noise variables to determine how much of a loss in power to expect from adjusting for covariates which were not related to outcome. Noise factors were generated as binary variables with a probability of 50%.

The parameters used in the simulation study can be found in Tables 1-3.

**Parameter values used in the simulation study**

**Table 1 – Parameter values used in simulation study for continuous outcomes***

| **Trial** | **Outcome** | **Prognostic covariates** | **Summary measure^¥^** | **Regression parameter** | **Correlation with outcome^#^** |
| --- | --- | --- | --- | --- | --- |
| FASTER | Oswestry disability index | Oswestry disability index at baseline | 46.4 (18.9) | 0.5 | 0.44 |
|  |  | Discectomy | 44% | -13.1 | -0.32 |
| MIST2 | Change in size of the patient’s pleural effusion from baseline to 7 days | Size of the pleural effusion at baseline | 42.6 (22.4) | -0.6 | -0.61 |
|  |  | Hospital-acquired infection | 12% | 5.1 | 0.12 |
|  |  | Large tube size | 13% | 4.3 | 0.16 |
|  |  | Drain present | 58% | 7.5 | 0.27 |
| MOSAIC | Epworth Sleepiness Score | Epworth Sleepiness Score at baseline | 8.0 (4.3) | 0.7 | 0.72 |
|  |  | Male | 78% | 0.6 | 0.01 |
|  |  | MRI received at baseline | 18% | -0.4 | -0.03 |
| TIME2 | Change in mean breathlessness over 42 days from baseline | Breathlessness at baseline | 58.5 (22.8) | -0.9 | -0.77 |
|  |  | Performance status 2-3 | 43% | 8.1 | 0.15 |
|  |  | Mesothelioma | 10% | -5.4 | 0.04 |

*The residual SDs used were: FASTER=18.2, MIST2=15.4, MOSAIC=2.7, TIME2=17.3

**^¥^** Summary measures are mean (SD) for continuous covariates (Oswestry disability index, size of pleural effusion, Epworth Sleepiness Score, breathlessness), and % for binary or categorical covariates (Discectomy, hospital-acquired infection, large tube size, drain present, male, MRI received, performance status 2-3, mesothelioma).

^#^  Correlations were calculated using Spearman’s rank correlation

**Table 2 – Parameter values used in simulation study for binary outcomes***

| **Trial** | **Outcome** | **Prognostic covariates** | **Summary measure^¥^** | **Log(odds ratio)** | **Correlation with outcome^#^** |
| --- | --- | --- | --- | --- | --- |
| AUGIB | Mortality in hospital | Clinical Rockall score | 2.6 (1.9) | 0.7 | 0.31 |
|  | Further bleeding in-hospital | Clinical Rockall score | 2.6 (1.9) | 0.3 | 0.17 |
|  | RBC transfusion in-hospital | Presence of shock | 37% | 0.9 | 0.23 |
|  |  | Haemoglobin concentration at baseline | 11.0 (3.2) | -0.8 | -0.72 |
| MIST2 | Need for surgery at 90 days | Size of the pleural effusion at baseline | 42.6 (22.4) | 0.03 | 0.24 |
|  |  | Large tube size | 13% | 0.8 | 0.06 |
| PROGRAMS | Sepsis-free survival up day 14 | Gestational age at birth | 28.2 (1.8) | 0.3 | 0.35 |
|  |  | Birth weight | 768 (193) | 0.004 | 0.38 |
|  | Mortality up to day 14 | Gestational age at birth | 28.2 (1.8) | 0.8 | -0.36 |
|  |  | Birth weight | 768 (193) | -0.007 | -0.38 |

*The log(intercepts) used were: AUGIB mortality=-4.5, further bleeding=-2.8, RBC transfusion=-0.8, MIST2=-3.5, PROGRAMS sepsis free survival=0.4, mortality=-2.5.

**^¥^** Summary measures are mean (SD) for continuous covariates (clinical Rockall score, Haemoglobin, size of pleural effusion, gestational age, birth weight), and % for binary or categorical covariates (presence of shock, large tube size).

^#^  Correlations were calculated using Spearman’s rank correlation

**Table 3 – Parameter values used in simulation study for time-to-event outcomes***

| **Trial** | **Outcome** | **Prognostic covariates** | **Summary measure^¥^** | **Log(hazard ratio)** | **Correlation with outcome^#^** |
| --- | --- | --- | --- | --- | --- |
| PBC | Time to death | Age | 50.0 (10.6) | 0.03 | 0.25 |
|  |  | Albumin level at baseline | 3.5 (0.4) | -1.0 | -0.28 |
|  |  | Log(bilirubin level at baseline) | 0.6 (1.0) | 0.9 | 0.50 |
|  |  | Histological stage 2** | 22% | 1.04 | 0.32 |
|  |  | Histological stage 3** | 38% | 1.36 | - |
|  |  | Histological stage 4** | 35% | 1.66 | - |
| RE01 | Time to death | WHO score of 2 | 25% | 1.0 | 0.16 |
|  |  | Tumour grade of 3 or 4 | 14% | 0.4 | 0.08 |
|  |  | Log(white cell count) | 2.1 (0.3) | 0.9 | 0.07 |

*Approximately 40% of patients in the PBC data had an event, and approximately 93% of patients in the RE01 data had an event.

**Hazard ratios for histological stages 2-4 are in comparison to histological stage 1. 5% of patients in the PBC trial had histological stage 1.

**^¥^** Summary measures are mean (SD) for continuous covariates, and % for binary or categorical covariates.

^#^  Correlations were calculated using Spearman’s rank correlation. Correlations were calculated between the prognostic covariate, and whether the patient experienced an event.

**Table 4 – Simulation results (continuous outcomes): mean treatment effect, standard error, and Z score**

|  | Unadjusted |  |  | Adjusted, prognostic |  |  | Adjusted, noise |  |  | Adjusted, prognostic and noise |  |  |
| --- | --- | --- | --- | --- | --- | --- | --- | --- | --- | --- | --- | --- |
| Study | Mean treatment effect | Mean SE | Z score | Mean treatment effect | Mean SE | Z score | Mean treatment effect | Mean SE | Z score | Mean treatment effect | Mean SE | Z score |
| MOSAIC – small treatment effect | 0.81 | 0.41 | 1.98 | 0.80 | 0.27 | 2.91 | 0.81 | 0.41 | 1.97 | 0.80 | 0.28 | 2.91 |
| MOSAIC – large treatment effect | 1.15 | 0.41 | 2.80 | 1.15 | 0.27 | 4.18 | 1.15 | 0.41 | 2.79 | 1.15 | 0.28 | 4.17 |
| MIST2 – small treatment effect | 5.77 | 2.94 | 1.96 | 5.77 | 2.15 | 2.69 | 5.76 | 2.96 | 1.94 | 5.76 | 2.16 | 2.66 |
| MIST2 – large treatment effect | 8.35 | 2.94 | 2.84 | 8.36 | 2.15 | 3.89 | 8.34 | 2.96 | 2.82 | 8.36 | 2.16 | 3.87 |
| TIME2 – small treatment effect | 10.34 | 5.20 | 1.99 | 10.32 | 3.42 | 3.01 | 10.37 | 5.28 | 1.96 | 10.32 | 3.47 | 2.97 |
| TIME2 – large treatment effect | 14.89 | 5.20 | 2.86 | 14.82 | 3.42 | 4.33 | 14.88 | 5.28 | 2.82 | 14.81 | 3.47 | 4.27 |
| FASTER – small treatment effect | 4.82 | 2.45 | 1.97 | 4.80 | 2.06 | 2.34 | 4.83 | 2.46 | 1.96 | 4.80 | 2.07 | 2.32 |
| FASTER – large treatment effect | 6.87 | 2.45 | 2.80 | 6.87 | 2.06 | 3.34 | 6.87 | 2.46 | 2.79 | 6.87 | 2.07 | 3.33 |

**Table 5 – Simulation results (binary outcomes): mean treatment effect, standard error, and Z score**

|  | Unadjusted |  |  | Adjusted, prognostic |  |  | Adjusted, noise |  |  | Adjusted, prognostic and noise |  |  |
| --- | --- | --- | --- | --- | --- | --- | --- | --- | --- | --- | --- | --- |
| Study | Mean treatment effect | Mean SE | Z score | Mean treatment effect | Mean SE | Z score | Mean treatment effect | Mean SE | Z score | Mean treatment effect | Mean SE | Z score |
| PROGRAMS (sepsis) – small treatment effect | -0.48 | 0.24 | -1.97 | -0.60 | 0.27 | -2.18 | -0.48 | 0.25 | -1.97 | -0.60 | 0.28 | -2.18 |
| PROGRAMS (sepsis) – large treatment effect | -0.68 | 0.24 | -2.77 | -0.84 | 0.28 | -3.05 | -0.68 | 0.25 | -2.77 | -0.85 | 0.28 | -3.05 |
| PROGRAMS (mortality) – small treatment effect | -1.08 | 0.54 | -1.99 | -1.18 | 0.57 | -2.07 | -1.10 | 0.55 | -2.00 | -1.20 | 0.58 | -2.07 |
| PROGRAMS (mortality) – large treatment effect | -1.68 | 0.67 | -2.50 | -1.81 | 0.70 | -2.59 | -1.70 | 0.68 | -2.51 | -1.85 | 0.71 | -2.60 |
| MIST2 (surgery) – small treatment effect | -0.80 | 0.41 | -1.94 | -0.86 | 0.43 | -2.00 | -0.81 | 0.42 | -1.94 | -0.88 | 0.44 | -2.00 |
| MIST2 (surgery) – large treatment effect | -1.29 | 0.48 | -2.71 | -1.38 | 0.49 | -2.78 | -1.31 | 0.48 | -2.71 | -1.40 | 0.50 | -2.79 |
| AUGIB rebleed – small treatment effect | -0.55 | 0.28 | -1.99 | -0.57 | 0.28 | -2.02 | -0.56 | 0.28 | -1.99 | -0.58 | 0.28 | -2.02 |
| AUGIB rebleed – large treatment effect | -0.84 | 0.30 | -2.79 | -0.87 | 0.31 | -2.83 | -0.85 | 0.30 | -2.80 | -0.87 | 0.31 | -2.83 |
| AUGIB rbc – small treatment effect | -0.33 | 0.17 | -1.97 | -0.67 | 0.24 | -2.77 | -0.33 | 0.17 | -1.97 | -0.67 | 0.24 | -2.77 |
| AUGIB rbc – large treatment effect | -0.45 | 0.17 | -2.71 | -0.91 | 0.24 | -3.73 | -0.46 | 0.17 | -2.71 | -0.92 | 0.25 | -3.73 |
| AUGIB mortality – small treatment effect | -0.62 | 0.31 | -1.97 | -0.70 | 0.33 | -2.10 | -0.62 | 0.32 | -1.97 | -0.71 | 0.34 | -2.09 |
| AUGIB mortality – treatment large | -0.95 | 0.35 | -2.73 | -1.06 | 0.37 | -2.88 | -0.95 | 0.35 | -2.73 | -1.07 | 0.37 | -2.88 |

**Table 6 – Simulation results (time-to-event outcomes): mean treatment effect, standard error, and Z score**

|  | Unadjusted |  |  | Adjusted, prognostic |  |  | Adjusted, noise |  |  | Adjusted, prognostic and noise |  |  |
| --- | --- | --- | --- | --- | --- | --- | --- | --- | --- | --- | --- | --- |
| Study | Mean treatment effect | Mean SE | Z score | Mean treatment effect | Mean SE | Z score | Mean treatment effect | Mean SE | Z score | Mean treatment effect | Mean SE | Z score |
| RE01 – small treatment effect | -0.23 | 0.11 | -2.01 | -0.28 | 0.11 | -2.48 | -0.23 | 0.11 | -2.01 | -0.29 | 0.12 | -2.48 |
| RE01 – treatment large | -0.32 | 0.11 | -2.76 | -0.39 | 0.12 | -3.38 | -0.32 | 0.11 | -2.77 | -0.39 | 0.12 | -3.38 |
| PBC – small treatment effect | -0.37 | 0.19 | -1.91 | -0.53 | 0.20 | -2.65 | -0.37 | 0.19 | -1.91 | -0.53 | 0.20 | -2.64 |
| PBC – treatment large | -0.56 | 0.20 | -2.76 | -0.78 | 0.21 | -3.74 | -0.56 | 0.20 | -2.76 | -0.79 | 0.21 | -3.73 |

**References**

1. Kahan BC, Morris TP. Reporting and analysis of trials using stratified randomisation in leading medical journals: review and reanalysis. BMJ. 2012;345:e5840. Epub 2012/09/18.

2. Bender R, Augustin T, Blettner M. Generating survival times to simulate Cox proportional hazards models. Stat Med. 2005;24(11):1713-23. Epub 2005/02/23.
